# Transcript levels of keratin 1/5/6/14/15/16/17 as potential prognostic indicators in melanoma patients

**DOI:** 10.1038/s41598-020-80336-8

**Published:** 2021-01-13

**Authors:** Wei Han, Chan Hu, Zhao-Jun Fan, Guo-Liang Shen

**Affiliations:** 1 Department of Burn and Plastic Surgery, The First Affiliated Hospital of Soochow University, No. 188 Shizi Street, Suzhou, 215000 People’s Republic of China; 2 Department of Surgery, Soochow University, Suzhou, 215000 People’s Republic of China; 3 Institute of Photomedicine, Shanghai Skin Disease Hospital, Tongji University School of Medicine, Shanghai, 200000 People’s Republic of China

**Keywords:** Cancer, Computational biology and bioinformatics, Genetics, Molecular biology, Biomarkers, Oncology

## Abstract

*Keratins *(*KRTs*), the intermediate filament-forming proteins of epithelial cells, are extensively used as diagnostic biomarkers in cancers and associated with tumorigenesis and metastasis in multiple cancers. However, the diverse expression patterns and prognostic values of *KRTs* in melanoma have yet to be elucidated. In the current study, we examined the transcriptional and clinical data of *KRTs* in patients with melanoma from GEO, TCGA, ONCOMINE, GEPIA, cBioPortal, TIMER and TISIDB databases. We found that the mRNA levels of *KRT1/2/5/6/8/10/14/15/16/17* were significantly differential expressed between primary melanoma and metastatic melanoma. The expression levels of *KRT1/2/5/6/10/14/15/16/17* were correlated with advanced tumor stage. Survival analysis revealed that the high transcription levels of *KRT1/5/6/14/15/16/17* were associated with low overall survival in melanoma patients. GSEA analysis indicated that the most involved hallmarks pathways were *P53* pathway, *KRAS* signaling, estrogen response early and estrogen response late. Furthermore, we found some correlations among the expression of *KRTs* and the infiltration of immune cells. Our study may provide novel insights for the selection of prognostic biomarkers for melanoma.

## Introduction

Skin cutaneous melanoma (SKCM) is one of the most deadly types of skin cancers as well as one of the most life-threatening malignancies damaging human health. Each year, melanoma accounts for over 80% of skin cancer-related deaths in the world^1^. The pathogenesis of SKCM, according to Clark model, gives the assumption that the progression from melanocytes to SKCM needs multiple steps, including formation of banal nevi, dysplastic nevi, melanoma in situ, and invasive melanoma^2^. Surgical resection is mostly preferred for the primary melanoma; however, metastatic melanoma is not sensitive with chemotherapy and radiotherapy^3^. Recently, immunotherapies and targeted therapies show promise for improving the prognosis of patients with advanced melanoma, such as immune checkpoint inhibitors that target *PD-L1* and *BRAF* inhibitor; but only a small amount of SKCM patients can benefit from it^4,5^. Therefore, early diagnosis in melanoma progression and identification of biomarkers is essential for the effective and rapid intervention and treatment of melanoma patients.

There are countless molecular mechanisms involved in the occurrence, development, and metastasis of SKCM. One such family of proteins believed to be closely related to the development and metastasis of SKCM is the keratin family. *Keratins *(*KRTs*), the intermediate filament (IF)-forming proteins of epithelial cells, are extensively used as diagnostic biomarkers in cancers^6,7^. *KRTs* family are closely associated with tumorigenesis and metastasis of multiple tumor types including lung^8^, breast^9^, and colon cancers^10^. The aberrant expression of keratin proteins has also been reported in melanoma. For example, overexpression of *KRT8* was observed in advanced melanomas^11^. However, the biological roles played by keratin proteins in melanoma is not fully understood, nor has there been any systematic attempt to assess the expression of these genes in melanoma or to assess how their expression relates to the clinical progression of this cancer.

In the present study, we analyzed publicly available gene and protein expression datasets to investigate the differential expression of *KRTs* and their relations with clinical parameters in SKCM patients. Furthermore, we also analyzed the predicted functions and pathways of the mutations in *KRTs*. The aim of the study was to provide insights into the molecular mechanisms of SKCM and to reveal potential novel therapeutic targets for this disease.

## Methods

### Data collection and processing

The gene expression dataset GSE46517 (Platform: Affymetrix Human Genome U133A Array) was obtained from the Gene Expression Omnibus (GEO) database (https://www.ncbi.nlm.nih.gov/geo/), which included 31 primary melanomas and 73 metastatic melanomas^12^. The TCGA SKCM dataset contains 475 tumor samples, including 104 primary and 371 metastatic samples, which included raw counts of RNAseq expression data and corresponding clinical information. Gene expression and clinical profiles of TCGA SKCM patients were downloaded from UCSC Xena (http://xena.ucsc.edu).

### Oncomine database

Then, transcriptional expression of *KRTs* in 20 common neoplasms was analyzed using the Oncomine online database (http://www.oncomine.com)^13^. Differentially expressed mRNAs were selected using the cut-off criteria: *P* = 0.01 (Student’s t-test), fold difference in expression 1.5, and differentially expressed gene rank ≤ 10%.

### Statistical analysis

Phenotype and transcriptional expression profiles in melanoma patients from TCGA were analyzed and displayed by using Graphpad Prism (version 8.0). We compared the T1-2 and T3-4 among *KRTs* in TCGA SKCM patients. *P* < 0.05 was considered statistically significant.

### Survival analysis

Gene Expression Profiling Interactive Analysis (GEPIA, http://gepia.cancer-pku.cn/) is a useful online tool that provide customizable and quick functionalities based on data from The Cancer Genome Atlas (TCGA; https://tcga-data.nci.nih.gov/tcga/) and the Genotype-Tissue Expression project (GTEx; https://www.gtexportal.org/home/index.html)^14^. GEPIA performs survival analysis based on gene expression levels, using log-rank test for the hypothesis evaluation. The horizontal axis (x-axis) represented time in days, and the vertical axis (y-axis) showed the probability of surviving or the proportion of people surviving. The lines presented survival curves of two groups. The blue curve represented the low expression of *KRTs* and the red curve represented the high expression of *KRTs.*

### The Human Protein Atlas database

The Human Protein Atlas (https://www.proteinatlas.org/) is a database of immunohistochemistry (IHC)‐based protein expression profiles in different cancers, normal tissue as well as cell lines^15^. *KRT* protein expression IHC images in clinical specimens of SKCM patients and normal skin tissues were obtained from this database.

### cBioPortal for cancer genomics dataset

The cBioPortal (http://cbioportal.org) is a straightforward online tool that can search for multidimensional cancer genomics datasets and provides access to data for more than 5000 tumor samples from over 20 cancer studies^16^. To study the *KRTs* mutation in SKCM, the cBioPortal database was used. Genomic alteration types and alteration frequency in SKCM were analyzed. The genomic alterations of *KRTs* included copy number amplification, mRNA upregulation, deep deletion, missense mutation with unknown significance, and so on.

### Protein–protein interaction (PPI) network construction

In the current study, STRING (http://string-db.org; version 11.0) was used to describe protein co-regulation of *KRTs* and measure functional interactions among nodes^17^. The interaction specificity score > 0.4 (the default threshold in the STRING database) was considered statistically significant. The Database for Annotation, Visualization and Integrated Discovery (DAVID, https://david.ncifcrf.gov/) can provide systematic and integrative functional annotation tools for users to investigate biological meaning behind the list of genes. Gene ontology (GO) analysis including the biological process (BP), cellular component (CC) and molecular function (MF) enrichment analysis were conducted for the selected *KRTs* by DAVID^18,19^, and then visualized in bubble chart. *P* value < 0.05 was considered statistically significant. Furthermore, GO:BP,CC,MF and KEGG functional enrichment were analyzed and plotted using ClueGO (version 2.5.3) and CluePedia (version 1.5.3)^20^.

### Gene set enrichment analysis (GSEA)

GSEA tool (version 2.10.1) was applied to predict associated up-regulated and down-regulated genes and the significantly changed pathways based on the expression profile from TCGA database^21^. In each separate analysis, Student’s-t-test statistical score is conducted in consistent pathways and the mean of the differentially expressed genes is calculated. A permutation test with 1000 times is utilized to detect the significantly involved hallmark pathways. The adj. P using Benjamini and Hochberg (BH) and false discovery rate (FDR) method by default is used to correct for the occurrence of false positive results. Significant involved genes are defined with an adj. *P* < 0.01 and FDR < 0.25.

## Results

### Transcriptional levels of KRTs in patients with SKCM

**Figure 1:**
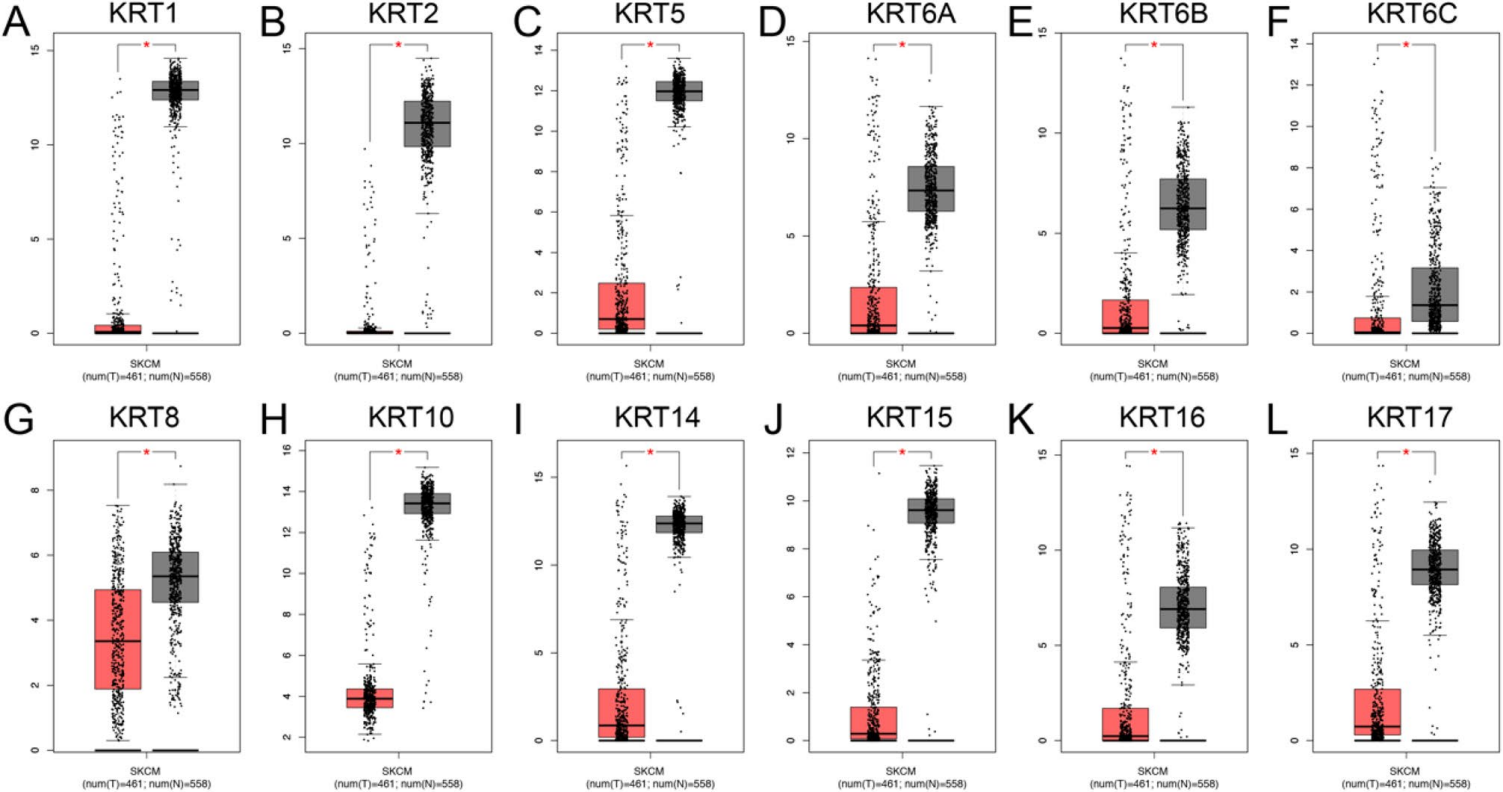
Transcriptional expression of distinct *KRTs* family members. (**A**–**L**) *KRTs* were down-regulated melanoma compared to normal skin (*P < 0.05).

After overlapping the GSE46517 and TCGA, we identified that *KRT1, KRT2, KRT5, KRT6A, KRT6B, KRT6C, KRT8, KRT10, KRT14, KRT15, KRT16* and *KRT17* were the most significantly differential expressed keratin family members between primary and metastatic melanoma, displayed in Supplementary Fig. 1 and Supplementary Fig. 2 in detail. *KRT1, KRT2, KRT5, KRT6A, KRT6B, KRT6C, KRT10, KRT14, KRT15, KRT16* and *KRT17* were all highly expressed in primary melanoma in both TCGA and GSE47517 cohort (*P* < 0.001); however, *KRT8* was overexpressed in metastatic melanoma in TCGA cohort. Next, we validated the expression of *KRT*s by using GEPIA. As shown in Fig. 1, all the *KRTs* are downregulated in melanoma compared to normal tissues.

### Expression pattern and survival analysis of KRTs in pan-cancer perspective

**Figure 2:**
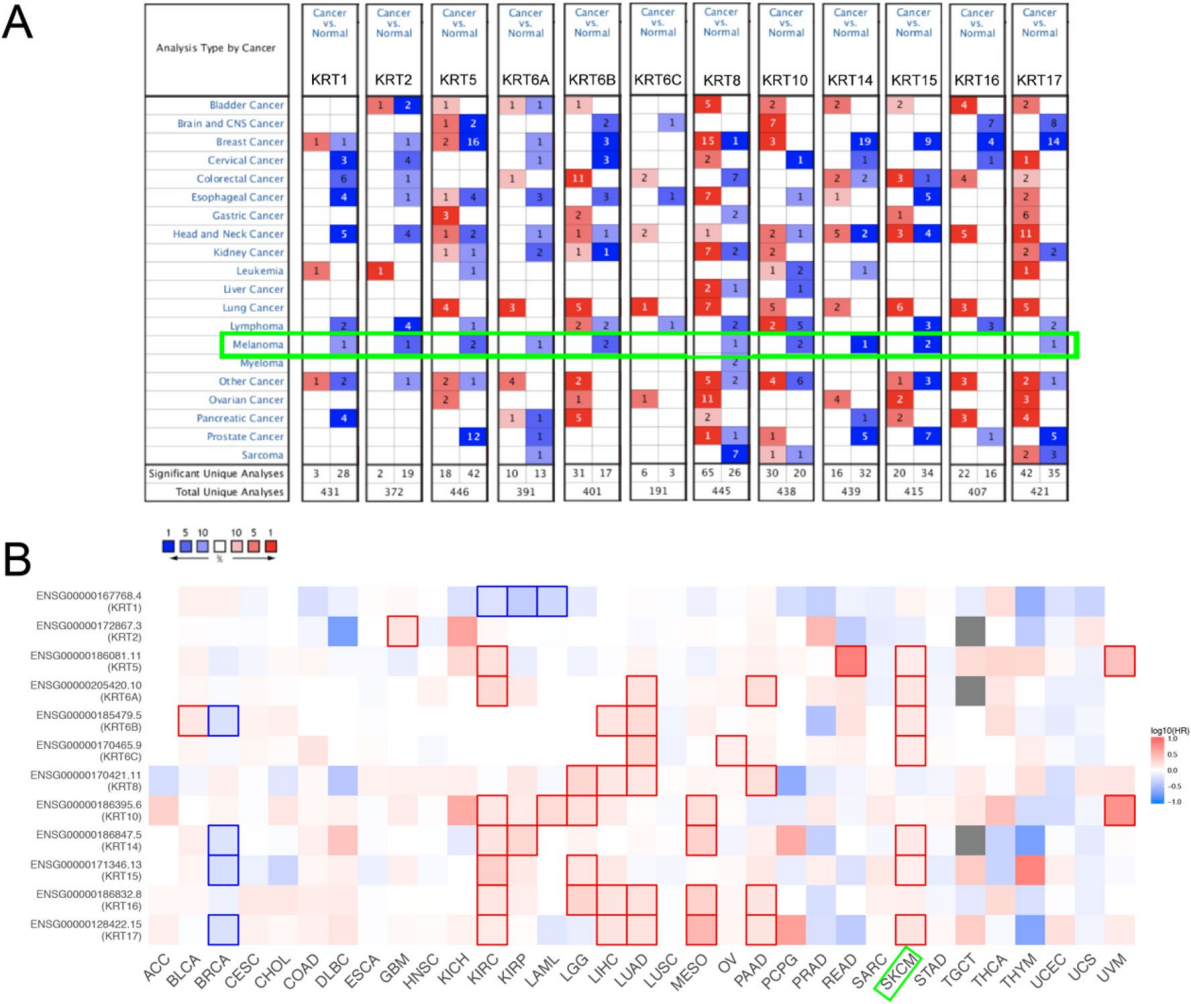
Expression level and survival analysis of *KRTs*. (**A**) Difference of transcriptional expression was compared by Students’ t-test. Cut-off of *P* value and fold change were as following: *P* value = 0.01, Fold Change = 1.5, gene rank = 10%, data type: mRNA. (**B**) Summary of hazard ratios (HR) illustrating cancer*-KRT* pairs with altered prognosis. (ACC, Adrenocortical Carcinoma; BLCA, Bladder Urothelial Carcinoma; BRCA, Breast Invasive Carcinoma; CESC, Cervical Squamous Cell Carcinoma and Endocervical Adenocarcinoma; CHOL, Cholangiocarcinoma; COAD, Colon Adenocarcinoma; DLBC, Lymphoid Neoplasm Diffuse Large B-cell Lymphoma; ESCA, Esophageal Carcinoma; HNSC, Head and Neck Squamous Cell Carcinoma; KICH, Kidney Chromophobe; KIRC, Kidney Renal Clear Cell Carcinoma; KIRP, Kidney Renal Papillary Cell Carcinoma; LAML, Acute Myeloid Leukemia; LGG, Brain Lower Grade Glioma; LIHC, Liver Hepatocellular Carcinoma; LUAD, Lung Adenocarcinoma; LUSC, Lung Squamous Cell Carcinoma; MESO, Mesothelioma; OV, Ovarian Serous Cystadenocarcinoma; PAAD, Pancreatic Adenocarcinoma; PCPG, Pheochromocytoma and Paraganglioma; PRAD, Prostate Adenocarcinoma; READ, Rectum Adenocarcinoma; SARC, Sarcoma; SKCM, Skin Cutaneous Melanoma; STAD, Stomach Adenocarcinoma; TGCT, Testicular Germ Cell Tumors; THCA, Thyroid carcinoma; THYM, Thymoma; UCEC, Uterine Corpus Endometrial Carcinoma; UCS, Uterine Carcinosarcoma; UVM, Uveal Melanoma).

As shown in Fig. 2 and Table 1, there was an apparent heterogeneity between different types of tumors. The mRNA expression levels of *KRT1, KRT2, KRT5, KRT6A, KRT6B, KRT8, KRT10, KRT14, KRT15 and KRT17* were significantly downregulated in patients with SKCM. In Riker’s dataset, the transcription levels of *KRT1* and *KRT2* in normal skin are higher than those in melanoma tissues, and those fold changes are − 6.313 and − 17.664, respectively^22^. The mRNA expression of *KRT5* in cutaneous melanoma decreases with a fold change of − 8.039(*P* < 0.005) in Riker’s dataset^22^ and − 71.976(*P* < 0.005) in Talantov’s datasets^23^. *KRT6A* is significantly downregulated in cutaneous melanoma, with fold changes of − 36.532 in Talantov’s dataset^23^. The transcriptional levels of *KRT6B* in cutaneous melanoma (fold change = − 19.758) significantly differ from those in the normal samples in Talantov’s dataset^23^. A similar trend is showed in *KRT10* in Riker’s^22^ and Talantov’s datasets^23^. In Riker’s dataset, the mRNA expression of *KRT8* and *KRT15* are found lower expressed in cutaneous melanoma with a fold change of − 2.794 (*P* < 0.005) and − 22.903 (*P* < 0.005)^22^. In Talantov’s dataset, *KRT14, KRT15* and *KRT17* are significantly downregulated in melanoma, with fold changes of − 234.928, − 219.782 and − 15.727, respectively^23^. Next, Kaplan–Meier curve as well as log-rank test analyses were performed to show the overall survival (OS). Results was shown graphically in Fig. 2B, seven members of *KRTs* were greatly related to OS in SKCM patients.

**Table 1:** Significant changes of *KRTs* expression in transcription level between SKCM and normal skin tissues (ONCOMINE).

Types of SKCM versus Normal skin	Fold Change	*P* value	t-test	Ref
***KRT1***				
Cutaneous melanoma versus normal	− 6.313	0.004	− 3.085	Riker Melanoma^22^
***KRT2***				
Cutaneous melanoma versus normal	− 17.664	4.96E−05	− 5.432	Riker Melanoma^22^
***KRT5***				
Cutaneous melanoma versus normal	− 8.039	0.004	− 3.11	Riker Melanoma^22^
Cutaneous melanoma versus normal	− 71.976	2.09E−11	− 11.142	Talantov Melanoma^23^
***KRT6A***				
Cutaneous melanoma versus normal	− 36.532	2.37E−05	− 6.518	Talantov Melanoma^23^
***KRT6B***				
Cutaneous melanoma versus normal	− 19.758	6.35E−09	− 9.366	Talantov Melanoma^23^
***KRT8***				
Cutaneous melanoma versus normal	− 2.794	0.003	− 4.348	Riker Melanoma^22^
***KRT10***				
Cutaneous melanoma versus normal	− 3.28	3.83E−04	− 4.182	Riker Melanoma^22^
Cutaneous melanoma versus normal	− 7.43	1.04E−05	− 8.863	Talantov Melanoma^23^
***KRT14***				
Cutaneous melanoma versus normal	− 234.928	1.52E−20	− 15.085	Talantov Melanoma^23^
***KRT15***				
Cutaneous melanoma versus normal	− 219.782	2.15E−18	− 17.132	Talantov Melanoma^23^
Cutaneous melanoma versus normal	− 22.903	0.002	− 3.707	Riker Melanoma^22^
***KRT17***				
Cutaneous melanoma versus normal	− 15.727	1.08E−05	− 5.769	Talantov Melanoma^23^

### Relationship between the mRNA levels of KRTs and the clinicopathological parameters of patients with SKCM

**Figure 3:**
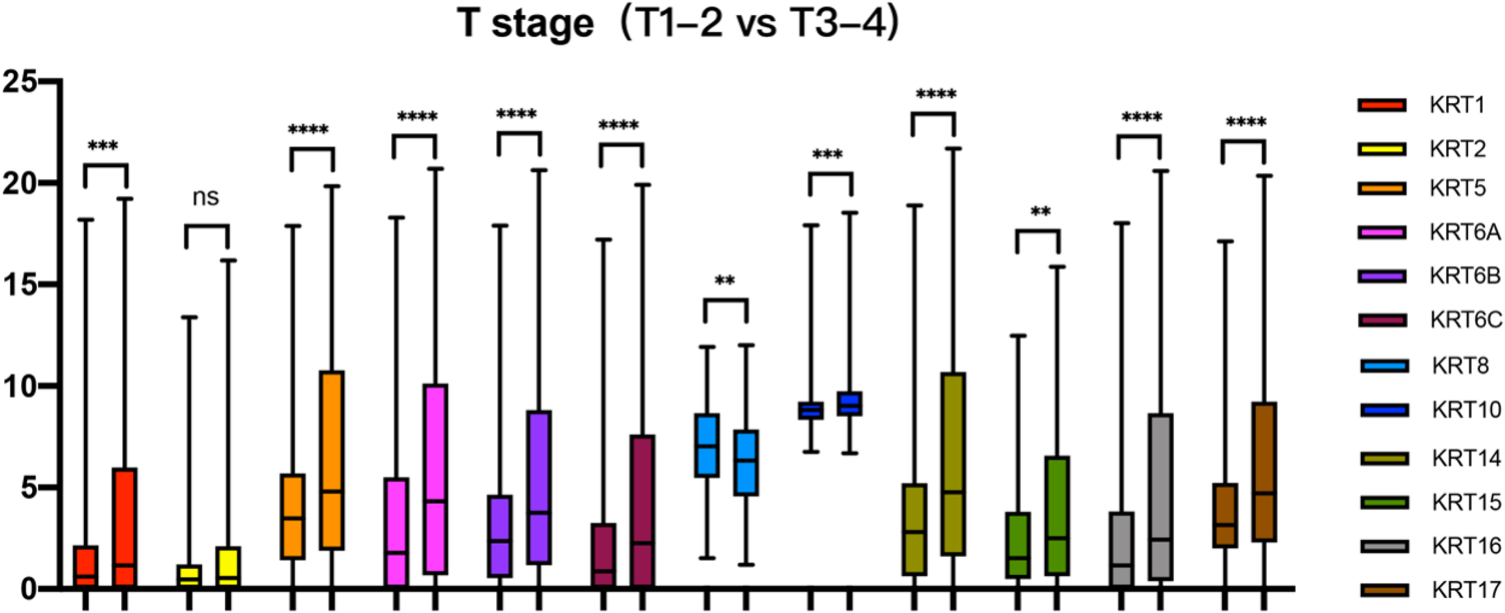
Relationship between transcriptional expressions of distinct *KRTs* family members and T stage of SKCM patients. Except *KRT2*, *KRT1/5/6/10/14/15/16/17* all showed significant correlations with SKCM patients (ns: no significance,*P < 0.05, **P < 0.01, ***P < 0.001).

In Fig. 3, except *KRT2*, elevated expression patterns of *KRT1, KRT5, KRT6A, KRT6B, KRT6C, KRT8, KRT10, KRT14, KRT15, KRT16* and *KRT17* were significantly associated with T stage (T1-T2 vs. T3-T4).

### Relationship between the mRNA levels of KRTs and the survival outcomes of patients with SKCM

**Figure 4:**
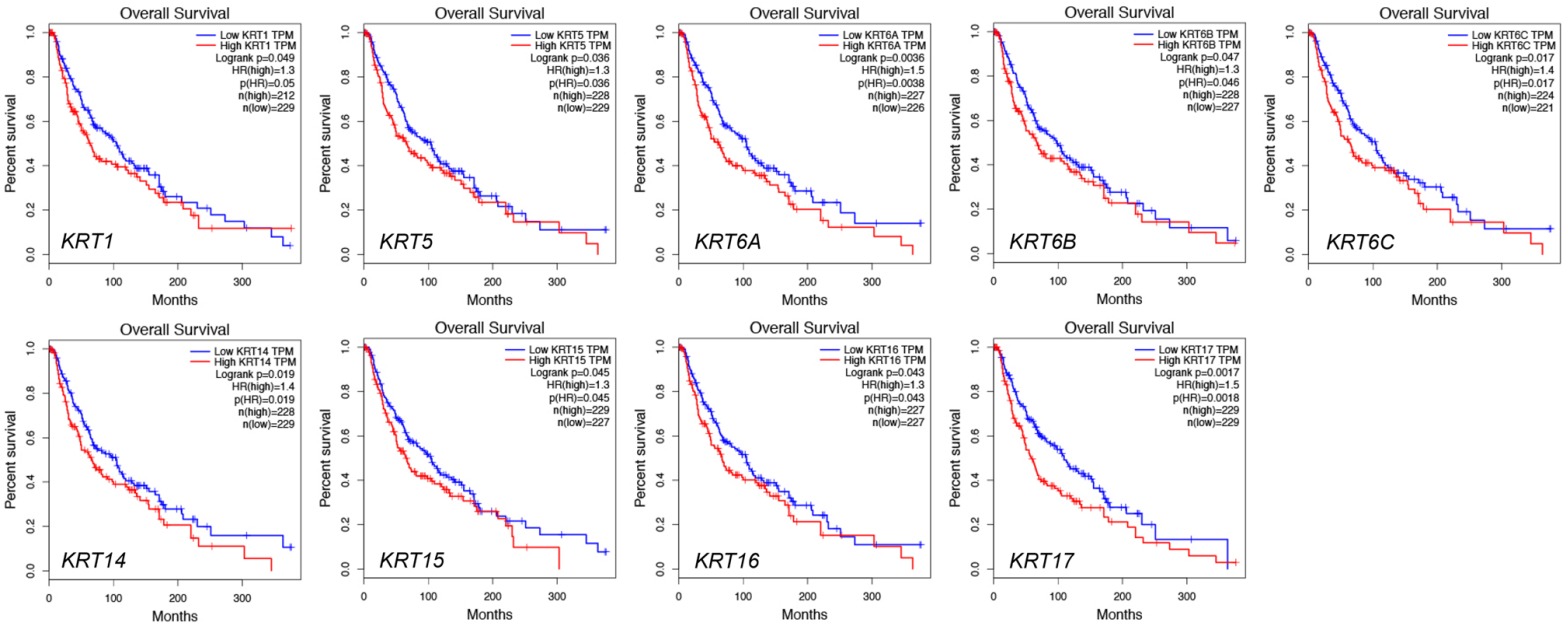
Kaplan–Meier survival analyses on differential *KRTs* expression groups with OS in the TCGA SKCM patients. High expression of *KRT1/5/6/14/15/16/17* were significantly correlated with poor OS.

We further explored the critical efficiency of *KRT*s in the survival of patients with SKCM. GEPIA was used to analyze the correlation between the mRNA levels of KRTs and the survival of patients with SKCM. The Kaplan–Meier curve and log-rank test analyses revealed that the increased *KRT1, KRT5, KRT6A, KRT6B, KRT6C, KRT14, KRT15, KRT16* and *KRT17* mRNA levels were significantly associated with the overall survival (OS) of melanoma patients (Fig. 4).

### Protein expression levels of KRTs in patients with SKCM

**Figure 5:**
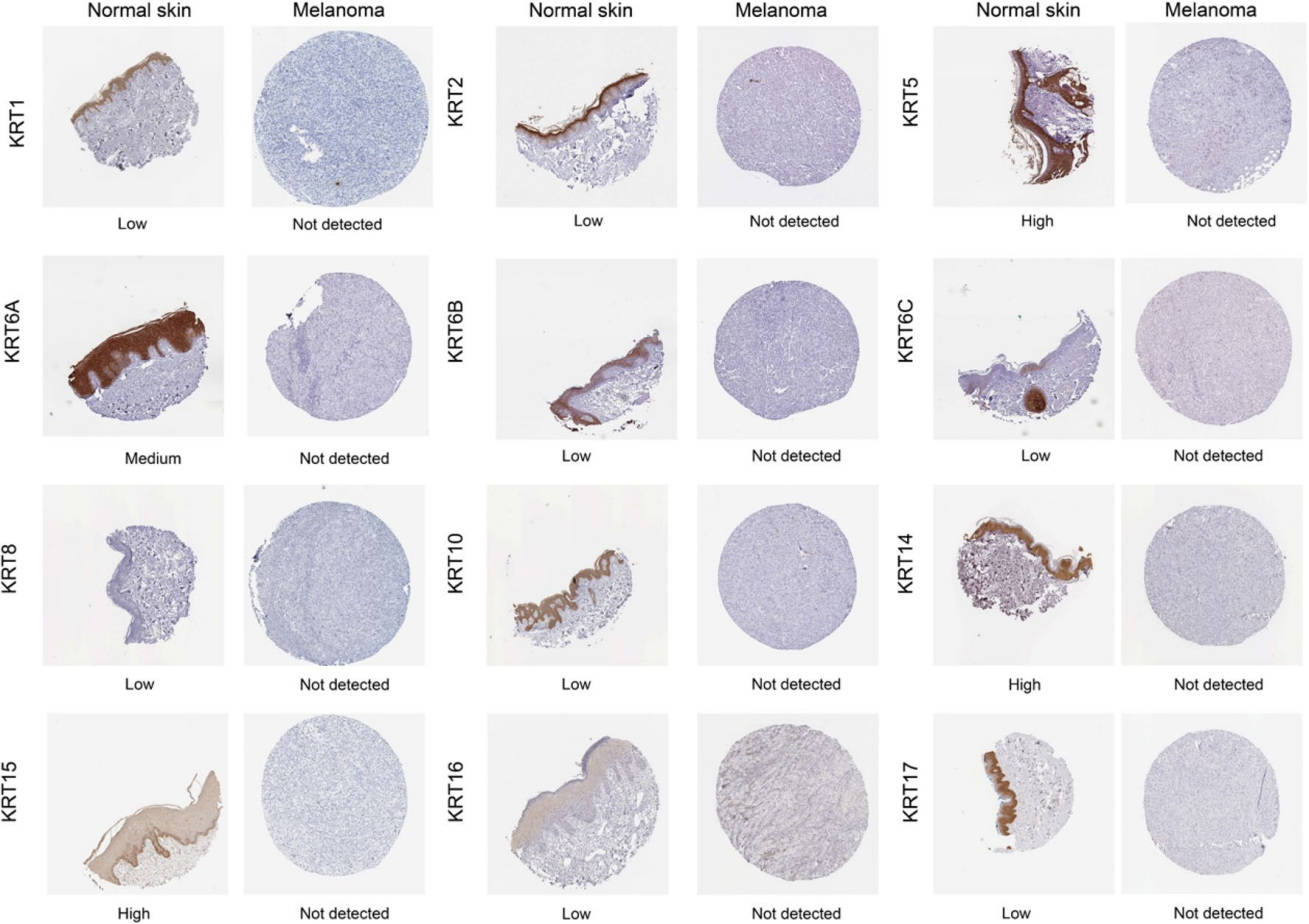
Immunohistochemical staining for protein expression of *KRT*s in tissues from patients with SKCM and normal tissues.

Next, to determine the differentially expression of *KRT* protein in melanoma tissues, IHC staining images for the *KRT* proteins in melanoma as well as normal skin tissues were obtained from the Human Protein Atlas database (Fig. 5). Consistent with the above results of *KRTs* mRNA expression, the results showed that *KRTs* protein levels were lower in melanoma than normal skin tissue.

### Genetic alterations and predicted interaction networks and signaling pathways of KRTs

**Figure 6:**
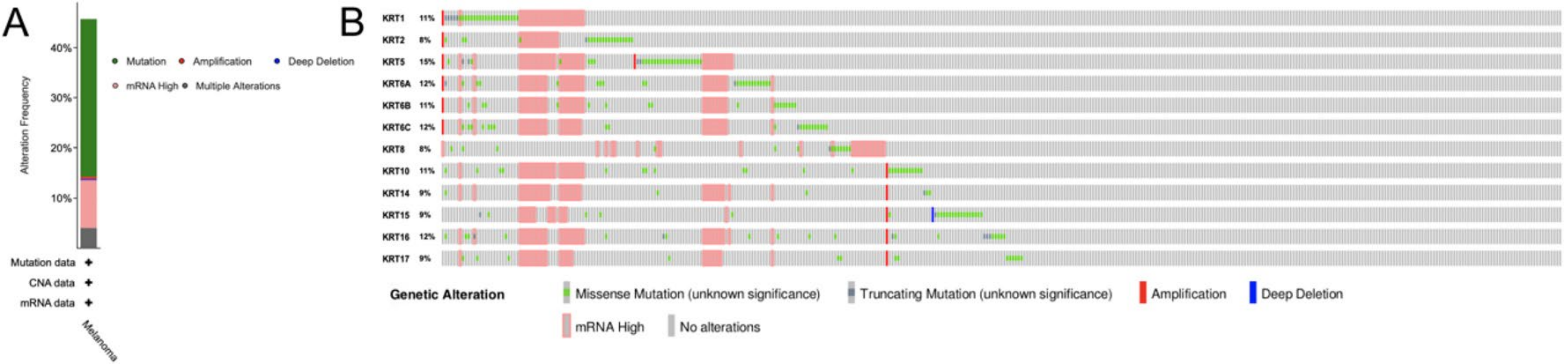
Genetic alterations in *KRT*s family members (cBioPortal). A visual summary of alteration based on a query of 12 *KRTs*, which was altered in 203 (45.72%) of 444 sequenced cases/patients.

High tumor mutation burden was reported to be a response biomarker for PD-1/PD-L1 blockade in melanoma^24^. Therefore, we analyzed the *KRTs* alterations and correlations by using the cBioPortal online tool for TCGA SKCM cohort. In the current study, different genetic alterations of *KRTs*, including missense mutation, truncating mutation, mRNA high and amplification, was shown in Fig. 6. *KRTs* were altered in 203 samples out of 444 SKCM patients (45.72%). *KRT5* (15%) was the most frequently altered genes among the *KRTs* genes, including amplification, fusion, and missense mutations. The missense mutation, which can change the polypeptide and therefore can change the function of the overall protein, is the most found mutation in all the *KRT*s.

**Figure 7:**
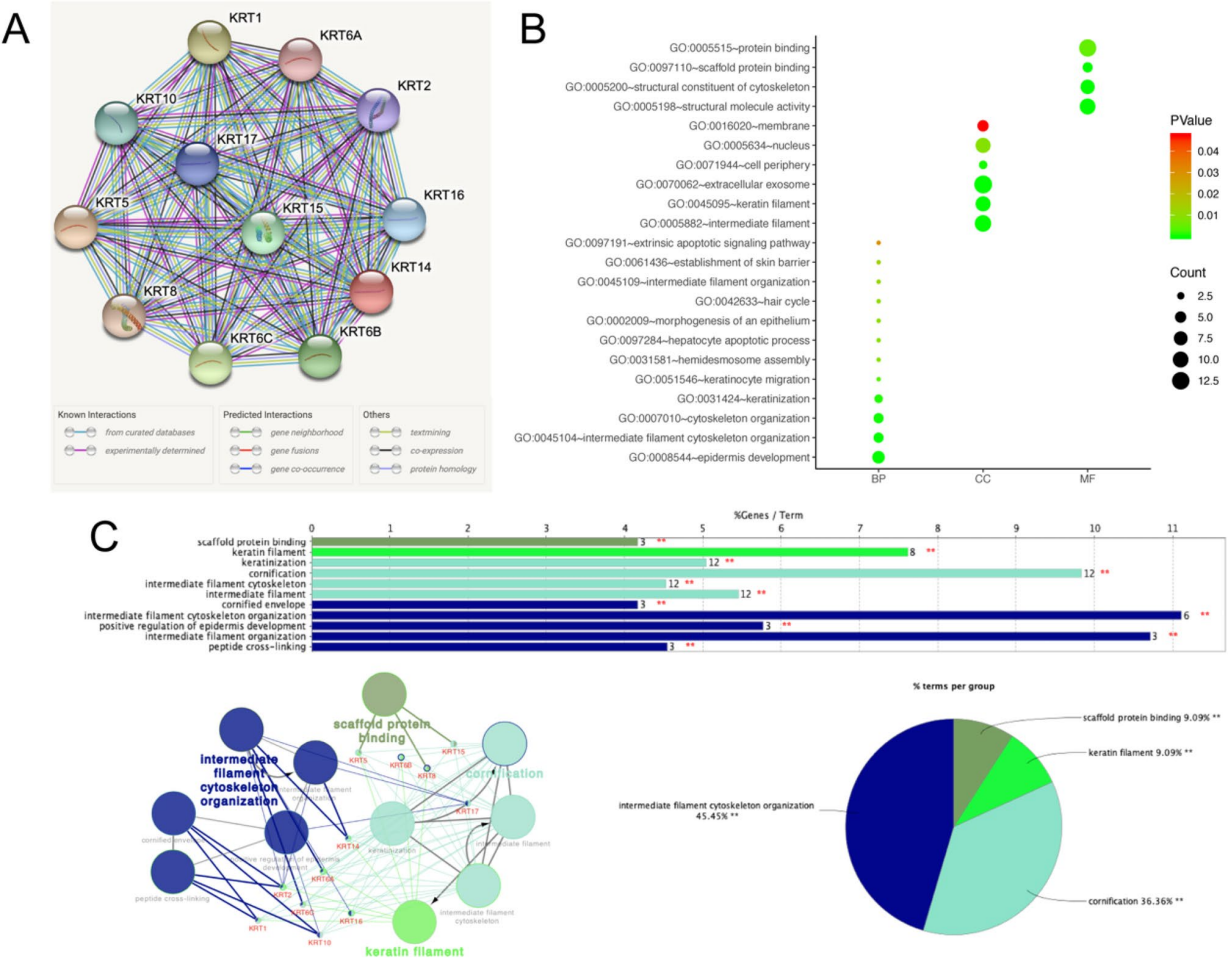
Functions enrichment and signaling pathways analysis of the mutations in *KRTs* in SKCM patients. (**A**) A PPI network analysis of differentially expressed *KRTs* with STRING was conducted to explore the potential interactions among them (**B**) Functional and pathway enrichment analyses of *KRTs* were performed using DAVID and visualized in bubble chart. (**C**) The functional annotation analysis of *KRTs* was constructed using ClueGO, a plug-in of Cytoscape.

Next, we conducted a PPI network analysis of differentially expressed *KRTs* with STRING to explore the potential interactions among them. As expected, several nodes of 12 and several edges of 66 were obtained in the PPI network (Fig. 7A). The functions of *KRTs* were predicted by analyzing Gene Ontology (GO) in the DAVID, visualized in bubble charts (Fig. 7B). GO enrichment analysis predicted the functional roles of target host genes on the basis of three aspects, including biological processes, cellular components, and molecular functions. We found that the biological processes of *KRTs* were significantly enriched in epidermis development, intermediate filament cytoskeleton organization, cytoskeleton organization, keratinization, keratinocyte migration and hepatocyte apoptotic process. Changes in cellular component were mostly enriched in intermediate filament, extracellular exosome, keratin filament and nucleus. As for molecular function, changes were primarily enriched in protein binding, structural molecule activity, structural constituent of cytoskeleton and scaffold protein binding. Then, ClueGO and CluePedia functional annotations revealed a network of the *KRTs* genes (Fig. 7C). The GO and Kyoto Encyclopedia of Genes and Genomes (KEGG) enrichment analysis of the *KRTs* are shown in different colors. The detailed functional notes and classification pie charts are listed in Fig. 7C; 45.45% terms belong to intermediate filament cytoskeleton organization, 36.36% to cornification, 9.09% to keratin filament, and 9.09% to scaffold protein binding.

**Figure 8:**
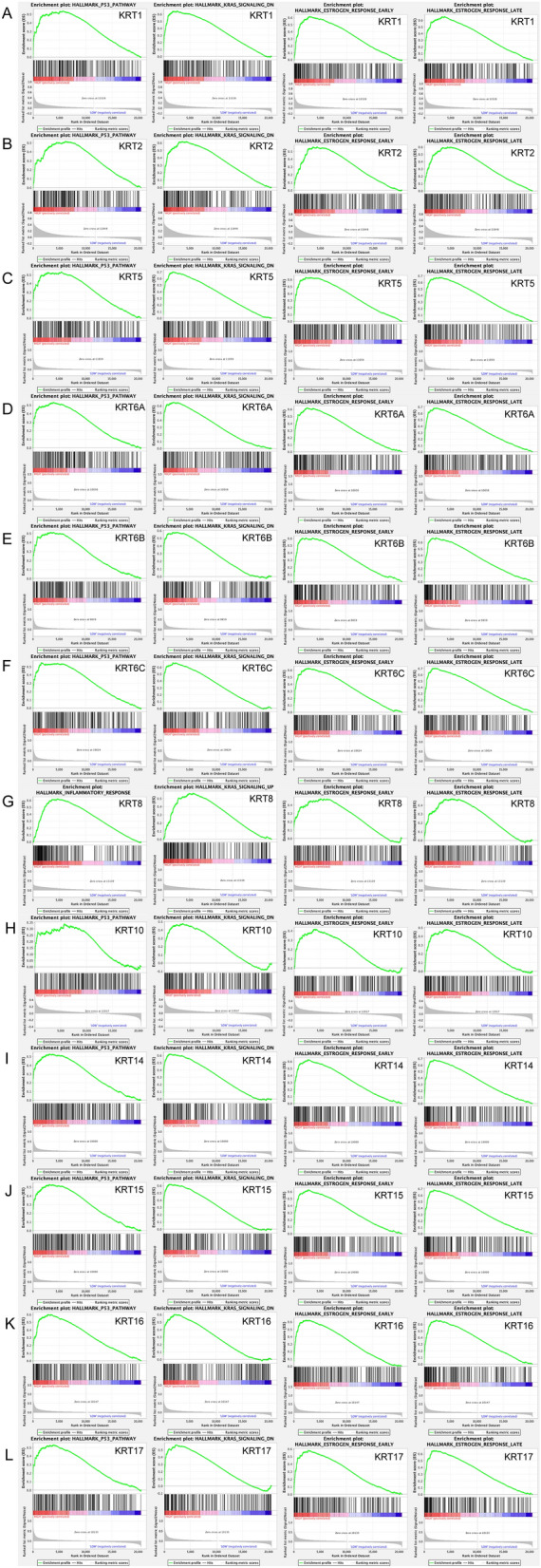
GSEA was used to perform hallmark signaling analysis in *KRTs*, respectively. A total of 100 significant genes were obtained from GSEA with positive and negative correlation. GSEA analysis, including *KRT1, KRT2, KRT5, KRT6A, KRT6B, KRT6C, KRT10, KRT14, KRT15, KRT16* and *KRT17* indicated that the most involved hallmarks pathways were *P53* pathway, *KRAS* signaling, estrogen response early and estrogen response late; whereas the most involved hallmarks of *KRT8* were inflammatory response, *KRAS* signaling, estrogen response early and estrogen response late.

Subsequently, a total of 100 significant genes were obtained from GSEA, and the genes with positive correlation were plotted. GSEA analysis, including *KRT1, KRT2, KRT5, KRT6A, KRT6B, KRT6C, KRT10, KRT14, KRT15, KRT16* and *KRT17* indicated that the most involved hallmarks pathways were *P53* pathway, *KRAS* signaling, estrogen response early and estrogen response late; whereas the most involved hallmarks of *KRT8* were inflammatory response, *KRAS* signaling, estrogen response early and estrogen response late. The details were illustrated in Fig. 8.

## Discussion

*Keratin *(*KRT*) is a family of intermediate filament proteins which expressed in various types of epithelial cells^25,26^. *KRTs* play significant roles in the occurrence, progression and metastasis of multiple cancers. For example, *KRT5, KRT14*, and *KRT17* overexpression are related to poor survival across different types of cancers, especially breast cancers^27,28^. However, reports about the function of keratin in melanoma are limited^29^. In this study, our results revealed that *KRTs* are heterogeneous in different types of tumors. According to the expression profiling analysis and survival analysis of TCGA, *KRTs* family members were significantly associated with the prognosis and clinical characteristic of SKCM patients.

Interestingly, we found that for all the differential expressed *KRTs* in the current study, they had the same trend that *KRTs* are highly expressed in primary melanoma while high expression of *KRTs* indicated poor prognosis. There are several assumptions to explain the results. Firstly, we think that melanoma in metastatic status might attract and activate more immune cells to fight against the tumor cells which leads to the lower expression; and because of the participation of immune cells, the survival outcomes of patients might get better. Secondly, the mutations in metastatic patients are much more complicated which might cause the increase of immunogenicity and activate the immune response contributing to a better prognosis. Thirdly, data from TCGA is not always accurate, therefore we need to further validate the results based on functional analysis experiments and more cases from prospective cohort studies in the future.

As a member of the keratin family, *KRT1* exists in the upper layer of the epidermis and the surface of endothelial cells, and plays a key role in the structural integrity of the skin. *KRT1* can produce intermediate filaments to enhance skin strength in the upper layer of the epidermis^30^. Soudy et al.^31^ found that *KRT1* protein was high expressed in breast cancer and showed great potential in the development of anti-tumor drugs. In our study, GEO and TCGA datasets revealed that the expression of *KRT1* was higher in primary melanoma than in metastatic melanoma. *KRT1* expression was significantly correlated with the clinical characteristics of the patients with SKCM. Using the GEPIA, we determined the prognostic value of *KRT1* in patients with SKCM. A high *KRT1* expression was significantly associated with poor OS in melanoma patients.

*KRT2* is one of the most abundant structural proteins of the epidermis; however, its biological significance remains unclear^32^. Cui et al.^33^ reported that *KRT2* can promote melanin production by transfecting *KRT2* to alpaca melanocytes. In our report, the expression of *KRT2* in primary melanoma tissues was higher than that in metastatic tissues. We also demonstrated that *KRT2* expression was significantly correlated with tumor stage in patients with SKCM. However, the expression of *KRT2* did not show much correlation with OS in melanoma patients.

*KRT5* is specifically expressed in basal layer of epidermis and plays an important role in protecting epithelial cells^34^. Elevated expression of *KRT5* was detected in recurrent and metastasized melanoma cell. In addition, researchers believed that *KRT5* participated in melanoma cell structure, metastasis and recurrence^29,35^, which is consistent with our findings. In the present study, we demonstrated that the expression of *KRT5* was lower in metastatic melanoma than in primary melanoma, and this expression was markedly correlated with tumor stage and T stage in patients with SKCM. High expression of *KRT5* could predict poor prognosis in melanoma patients.

*KRT6A* and *KRT6B* both play important roles in the collective migration of keratinocytes^36^. Previous studies revealed that *KRT6A* silencing can suppress cell invasion and metastasis of nasopharyngeal carcinoma^37^. *KRT6C* was found to be closely related to the prognosis and metastasis of lung adenocarcinoma^38^. However, few studies discuss the role of them in melanoma. We first found that *KRT6A/B/C* are overexpressed in primary melanoma compared to metastatic melanoma. Increased expression of *KRT6A/B/C* are all significantly associated with the poor prognosis in SKCM patients.

*KRT8* and *KRT18*, the hallmark keratin pair of all simple epithelia, usually collaborate with each other to work^39^. *KRT8* and *KRT18* both can regulate protein synthesis, participate in cell movement and inhibit apoptosis. Previous studies suggested that the abnormal expression of *KRT8/18* are associated with invasiveness and poor prognosis in cancers^40^. In melanoma, *KRT8/18* was thought to be related to increased invasiveness and metastasis. Safadi et al.^41^ reported that expression of *KRT8/18* in metastatic melanoma are higher than that of primary cutaneous and mucosal melanoma. In addition, some researchers co-transfected a low invasive human melanoma cell line with c-DNAs for *KRT8/18* to a more invasive resultant^42^. Interestingly, the increased expression of *KRT8/18* in adenocarcinoma is related with a better prognosis^43,44^. In our study, we found that the expression of *KRT8* was higher in metastatic melanoma than primary melanoma in TCGA cohort, whereas it showed converse results in GSE46517 cohort. As for *KRT18*, both TCGA and GEO datasets showed no difference in the expression of *KRT18*. Therefore, further confirmation is needed.

*KRT10*, expressed in the spinous and corneal layer^45^, is closely associated with hereditary skin diseases^46–48^. Chen et al.^49^ found that *KRT10* can increased tumor susceptibility of epithelial cells. However, few research showed its role in melanoma. In our study, we found that *KRT10* is highly expressed in primary melanoma than metastatic melanoma. Increased expression of *KRT10* is correlated with the T stage as well as the tumor stage in melanoma.

*KRT14* is usually found in keratinocytes of the basal layer. In nodular melanoma, *KRT14* was found strongly present in basal layer as well as in suprabasal cells^50^. Bilandzic et al.^51^ demonstrated that *KRT14* showed great invasive potential in ovarian cancer and can act as a novel target in anti-tumor therapies. Papafotiou et al.^52^ suggested *KRT14* played a pivotal role in regeneration and tumorigenesis in bladder cancer. In our study, high expression of *KRT14* is closely related to tumor stage and poor prognosis in melanoma patients, which may provide new clues for the diagnosis and treatment of malignant melanoma.

*KRT15* is found to be related to the development of many tumors, including breast and lung cancer^53–56^. In addition, *KRT15* was observed to be coordinately expressed with melanoma-associated chondroitin sulfate proteoglycan^57^. In our study, the expression of *KRT15* is higher in primary melanoma than in metastatic melanoma. *KRT15* also correlates with the tumor stage and poor survival in melanoma patients.

*KRT16,* located at chr.17q21.2, encodes for the type I cytoskeletal 16 protein^58^. Increased expression of *KRT16* is significantly associated with poor prognosis in squamous cell carcinoma^59^. In addition, *KRT16* contributes to the immune response to tumors and in tumor cell development^60^. Moreover, the levels of *KRT16* expression may discriminate metastasis from primary melanoma^61^. In the present study, we found that *KRT16* is overexpressed in primary melanoma compared to metastatic melanoma and showed significant difference in different T stage and pathological stage. Increased expression of KRT16 is associated with the poor prognosis in SKCM patients. Importantly, there was a negative correlation between the expression of *KRT16* and the infiltration of CD8^+^ T cells, CD4^+^ T cells, macrophages, neutrophils, and dendritic cells, which is consistent with previous findings and might provide insights into the immunotherapy of melanoma patients.

*KRT17* is mainly present in the epithelial appendages, such as hair follicles, sebaceous glands and other glands. *KRT17* was found to be overexpressed in several cancers, including breast cancer and cervical cancer^62^. In addition, *KRT17* can regulate proliferation of cancer cell, and induce the proliferation of squamous cell carcinoma cells^63^. Kung et al.^64^ found that the tumor suppressor protein P53 negatively regulates *KRT17* and repressed *KRT17* transcription. The same results were observed in our study. In the current study, we found that *KRT17* significantly overexpressed in primary melanoma than metastatic melanoma, and their expression levels were markedly correlated with the tumor stage of the SKCM patients. Interestingly, the high *KRT17* expression was significantly but inversely, correlated with poor OS in melanoma patients.

In this study, GO analysis showed that the biological processes of *KRTs* were significantly enriched in epidermis development, intermediate filament cytoskeleton organization, cytoskeleton organization, keratinization, keratinocyte migration and hepatocyte apoptotic process. GSEA analysis indicated that the most involved hallmarks pathways were *P53* pathway, *KRAS* signaling, estrogen response early and estrogen response late, which all played significant roles in the progression of the malignant phenotype of melanoma.

Our study is the first to investigate the possible prognostic utility of *KRTs* in SKCM. While the preceding discussion illustrates the potential involvement of *KRTs* in the development of many cancers and human diseases, it is noteworthy that little is known about their involvement in SKCM. In this study, we demonstrated the significant upregulation of *KRTs* in SKCM and its correlation with T stage and OS. Interestingly, we also showed that genetic alteration of *KRTs* is present in 45.72% of SKCM patients. However, further validation studies and prospective cohort studies are needed to verify these findings. Future research will explore the mechanistic differences between the *KRTs* in SKCM and other carcinomas.

## Conclusion

In conclusion, our study is the first to demonstrate that *KRT1/2/5/6/8/10/14/15/16/17* expression is elevated in primary melanoma compared with metastatic, and that high *KRT1/5/6/14/15/16/17* mRNA levels predict poor prognosis. These novel findings not only shed light on the molecular alterations in SKCM but also provide the foundation for further research in this area.

## Supplementary information

Supplementary Figure 1.

Supplementary Figure 2.
