# Supplementary figures and images for: Transcript levels of keratin 1/5/6/14/15/16/17 as potential prognostic indicators in melanoma patients
Source: Sci Rep. 2021 Jan 13;11:1023. doi: 10.1038/s41598-020-80336-8 (PMC7806772; doi:10.1038/s41598-020-80336-8)

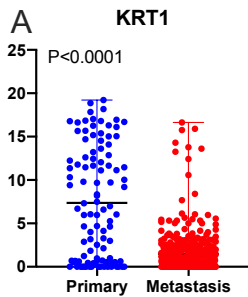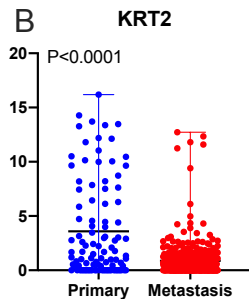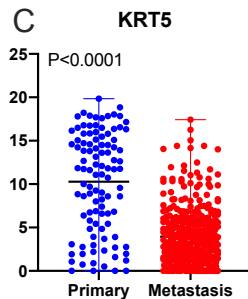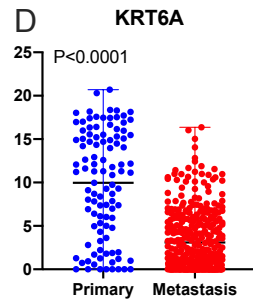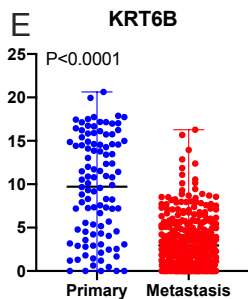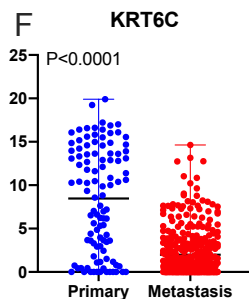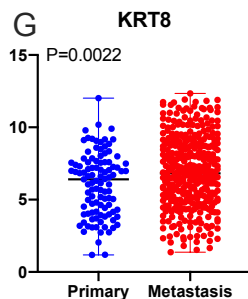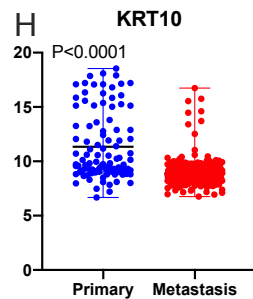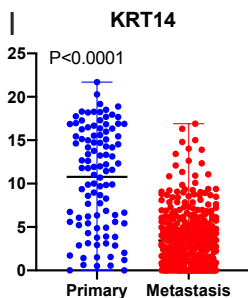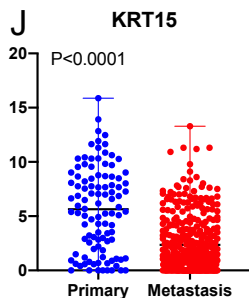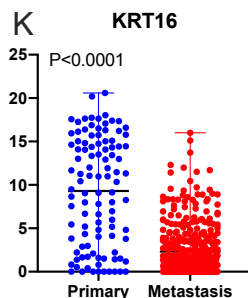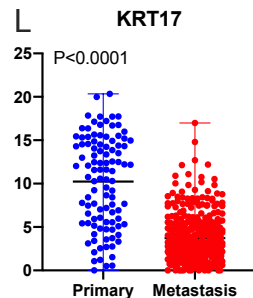

Supplement: Supplementary file 1 — Supplementary Figure 1. [file 41598_2020_80336_MOESM1_ESM.pdf]

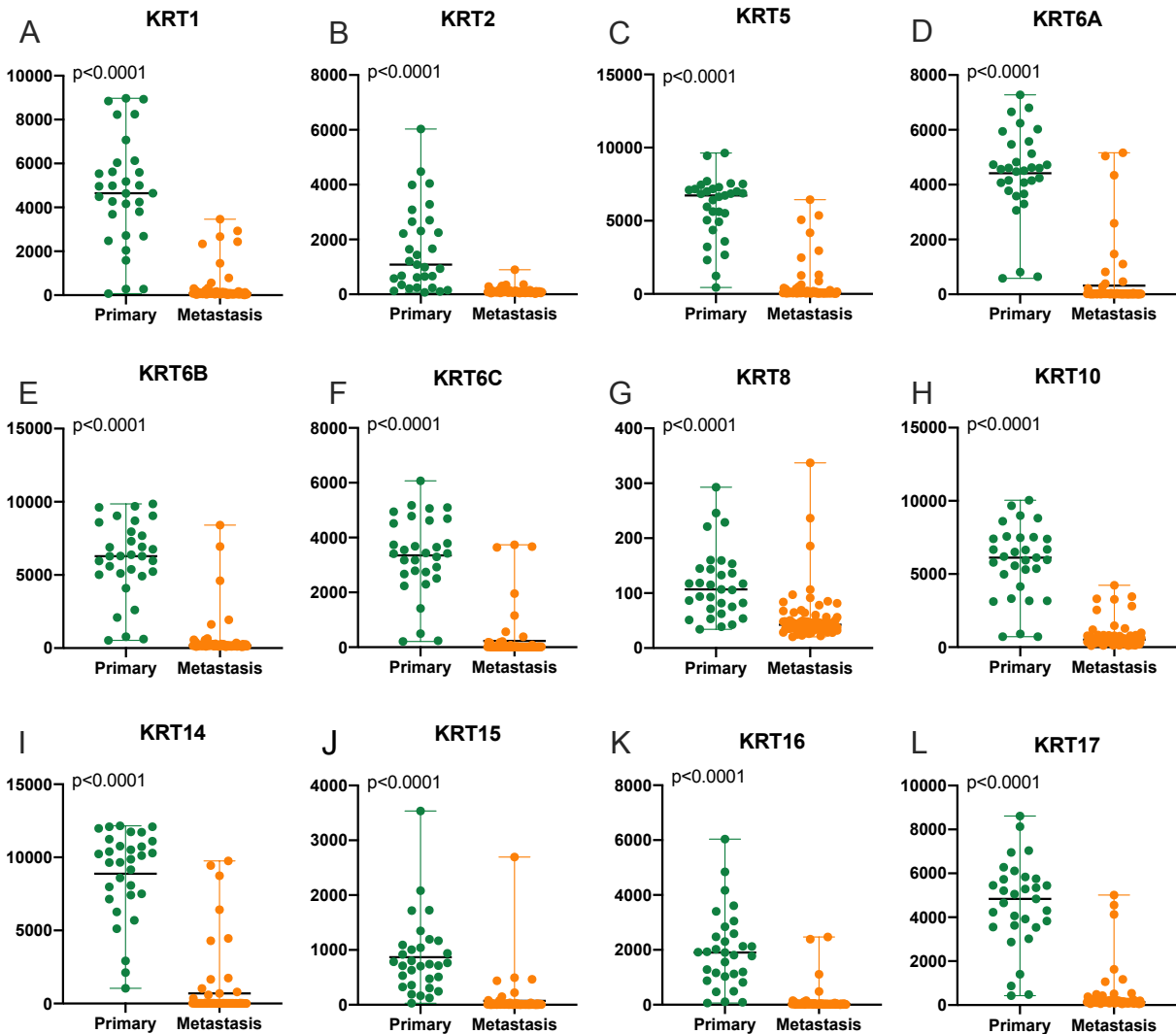

Supplement: Supplementary file 2 — Supplementary Figure 2. [file 41598_2020_80336_MOESM2_ESM.pdf]
